# 

**Published:** 1878-06

**Authors:** 


### CONTENTS:

ARTICLE I.—Lesions of the Trifacial (Especially Facial Neuralgia,) Resulting from Diseases of the Dental Organs and Adjacent Parts. By Dr. H. Goodwillie, M. D., D. D. S. .... 49
ARTICLE II.—The Liquefaction of Gases .... 63
ARTICLE III.—Some of the Causes of the Decay of the Teeth of Americans. By Prof. Hodgkin .... 71
ARTICLE IV.—Care of Children's teeth. By Dr. Klump .... 77
ARTICLE V.—The Use of Dissimilar Metals in Filling a Tooth. By Dr. A. W. Harlan .... 82
ARTICLE VI.—Chloride of Calcium .... 85
ARTICLE VII.—Dental Association Meetings:
California .... 85
South Carolina .... 86
American Dental .... 86
Southern Dental .... 87

### EDITORIAL, ETC.

The Newest of our Creeds .... 87

The Dental and Oral Science Magazine .... 89

### MONTHLY SUMMARY.

Gelseminum Sempervirens in Neuralgia .... 90

Effects of Lymph on Cicatrization .... 91

Absurd.—The Source of Artificial Teeth .... 91

The Elimination of Mercury .... 92

Relation of Brain Weight to Mental Ability .... 92

Curious Development of Teeth .... 92

Statistics of Insanity .... 93

Dangers from Salicylic Acid .... 93

Replantation of Teeth .... 93

Treatment of Earache .... 94

Food and the Human Body .... 95

Salicylic Acid as a Dentifrice .... 95

Tubercular Ulcer of the Tongue .... 96

The Cell and Protoplasm .... 96

